# Linking perception and cognition

**DOI:** 10.3389/fpsyg.2013.00144

**Published:** 2013-03-22

**Authors:** Arnon Cahen, Michela C. Tacca

**Affiliations:** ^1^Department of Philosophy, Haifa UniversityHaifa, Israel; ^2^Department of Philosophy, Heinich-Heine University DüsseldorfDüsseldorf, Germany

As our primary mode of contact with the world, perception is the causal and informational foundation for our higher cognitive functions—it guides our thinking about and acting upon the world. It is therefore unsurprising that so much empirical and theoretical research is devoted to the study of the complex interrelations between perception and cognition. Nor is it surprising that such research spans traditional disciplinary boundaries and attracts the interest and efforts of researchers from the full spectrum of the cognitive sciences, psychology, neuroscience, philosophy, and others. This Research Topic aims to contribute to this expansive research project—the exploration of the perception/cognition interface—while respecting its essentially interdisciplinary character.

Given that perception is *the* input to cognition, the two systems must be able to “talk” to each other; at the very least, information carried by perception must be of a form adequate to be “taken in” by our various cognitive systems. The central questions of this Research Topic surround the nature of their “communication.” In particular, we ask: what kinds of structural relations must hold between perceptual and cognitive representations for such communication to be possible? To what extent, if at all, is it a “dialog?” And, what mechanisms mediate the transitions between the two systems? As the papers here included exemplify, all these questions are open to exploration from within a variety of different disciplines and perspectives.

The question on the structural relation between perception and cognition is addressed by Tacca (2011) who argues that there are important structural similarities between early vision and higher cognitive systems underlying active empirical thought—in particular, she argues that both involve systematic representations. Given that systematicity is a central feature of representations employing conceptual capacities, the systematicity of early vision implies that it might count as an early type of conceptual representation. Such a view helps explain how perceptual representations can inform our thoughts about the world. Raftopoulos (2011), on the other hand, proposes that *early* vision might not involve cognition-like structural properties, though he continues to argue that *late* vision is cognitively penetrable and not purely visual. Nonetheless, unlike representations involved in higher cognition, late vision representations do not support inferences and discursive reasoning. This view is shared by Tacca, who, for this reason, considers perceptual representations as an early type of conceptual representations.

Roth and Franconeri (2012) suggest a further property shared between vision and high-level cognition: asymmetry. The asymmetry in question is found in spatial language: in sentences like “A is above B,” A and B are assigned different roles: the first is the “figure” and the other “ground.” Similarly, they argue, in vision, the perception of A being above B may be asymmetrically encoded such that the one is at the “spotlight” of attention, and has a certain, behaviorally manifest, priority over the other. They further implicate spatial attention as the mechanism underlying this asymmetric encoding.

Potter (2012) explores which mechanism might mediate between perception and cognition by reviewing evidence for conceptual short-term memory—a mental buffer in which perceptual stimuli and their related concepts come together for a brief time. This allows for the identification of meaningful patterns and structures. Potter considers perception and cognition as roughly continuous—as different processing stages operating on the same representational format.

Another possible mechanism linking perception and cognition is attention. Brown et al. (2011) expand upon the thought that attention mediates spatial visual perception and investigate its possible role in biasing proprioceptive signals in the motor system. They investigate further possible similarities between the attentional mechanisms underlying spatial perception and those operating on proprioceptive inputs.

Goldstone et al. (2011) investigate a specific perceptual mechanism that might be influenced by cognition: the extraction of distant similarities. They suggest that high-level cognition directs attention so as to modulate what they call “categorical perception.” They argue that perception is plastic and that “it is not just perceptual sensitivities that are driving the categories, but rather the acquired categories are also driving perceptual sensitivities, (p. 385).” They further emphasize: “There is little, if any, gap between perception and high-level cognition because perceptual systems adapt to fit the needs of high-level cognition, (p. 385).” Perception is suffused by cognition. Goldstone et al.'s account is based on the so-called feature-based approach to concepts; namely, the idea that concepts are composed of perceptual stimuli.

Stöckle-Schobel (2012) reviews the success of the feature-based approach to concept acquisition (see, Schyns and Rodet, 1997; Goldstone and Barsalou, 1998; Barsalou, 1999; Goldstone et al., 2011). He argues that if a feature-based theory of concept learning is to be successful it must address a number of philosophical challenges originally posed by Fodor (1981, 2008) against the possibility of acquiring genuinely novel concepts. He argues that these challenges have not been fully met by the feature-based approach, and provides recommendations for how proponents of such an approach might eventually meet them.

Tanaka et al. (2012) further explore the role of categorization in the process of perceptual recognition. They do so via an exploration of the atypicality bias—the finding that objects that are equally similar to two examples of the same category will be perceived as more similar to the more atypical of the two. This suggests that perceptual recognition of some object is influenced by the density and organization of exemplars in the object's category space.

Lebrecht et al. (2012) focus on the affective content of perceptual representations. They argue that even the very subtle valence, or micro-valence, of an object has a significant unconscious influence on how those objects are perceptually represented and recognized. They argue that such effects are so robust that affect can be understood as a straightforwardly perceptible property of objects.

Finally, Mroczko-Wąsowicz and Werning (2012) provide a sensory-motor account of synesthesia that considers the role of top–down associations in shaping the synesthetic experience. Particularly, they argue that swimming style synesthesia can be seen as a case of hyperbinding that—unlike normal binding (Barsalou, 2008)—combines sensory attributes that do not normally form a concept frame. This interpretation calls for a larger integration between visuo-motor representations and conceptual representations.

Taken together the papers in “Linking Perception and Cognition” offer a wide perspective of the theoretical and empirical research on the nexus between how we perceive the world and how we think about it. These papers provide useful guides toward better understanding the connections that make our mental life so rich, indeed, that make it possible at all.
